# Visual Snow Syndrome: Seeing Snowflakes, a Unique Visual Phenomenon Following Mild Traumatic Brain Injury

**DOI:** 10.1002/ccr3.73289

**Published:** 2026-08-07

**Authors:** Sarah Matthews, Nathan Evanson, Katherine Hogan, Brad Kurowski

**Affiliations:** ^1^ Department of Pediatric Rehab Medicine Cincinnati Children's Hospital Medical Center Cincinnati Ohio USA; ^2^ Department of Pediatrics University of Cincinnati College of Medicine Cincinnati Ohio USA; ^3^ Division of Ophthalmology Cincinnati Children's Hospital Medical Center Cincinnati Ohio USA

**Keywords:** case report, mild traumatic brain injury, sports related concussion, visual changes, visual snow syndrome

## Abstract

Visual Snow Syndrome (VSS) can occur after a variety of triggers including mild traumatic brain injury (mTBI). Rehabilitation providers frequently evaluate patients following mTBIs. It is vital for rehabilitation providers to include VSS on their differential diagnosis to facilitate appropriate referrals to Ophthalmology for evaluation and diagnosis.

## 1 Introduction

This case of visual snow syndrome (VSS) following a sports related mild traumatic brain injury in an adolescent highlights a unique presentation of this visual phenomenon that can occur following a mild traumatic brain injury from a rehabilitation perspective, of which there is a lack of literature.

## 2 Case History/Examination

A 15‐year‐old male with no past medical or family history was referred to a physiatrist for a sport related mTBI. The patient sustained a mTBI 2 months prior to evaluation when he was tackled during a football game and the back of his helmet hit the ground. Initial symptoms, including headache and flashes in vision, worsened after returning to football practice 1 week later.

At the time of presentation, he was having weekly retroorbital headaches, worse at the end of the school day and described constant small bright flashes in his eyes. He had returned to full days of school and conditioning preseason workouts with the baseball team. The reported bright flashes were constant and did not worsen with headaches or activity. On exam, he had intact extraocular movements, no vertical or horizontal nystagmus, and no worsening of visual symptoms or headaches with eye testing. His neurologic exam was otherwise normal.

## 3 Differential Diagnosis, Investigations, and Treatment

He was referred to ophthalmology for further evaluation of his vision concerns and physical therapy to focus on sport specific activities. He was counseled on pacing both physical activity and school work, maintaining hydration consistently throughout the day and was cleared to return to non‐contact sports. He returned for additional follow up 7 weeks after initial evaluation following completion of a course of physical therapy. His ImPact testing [1] scores were average to above average (see Table 1), felt to be consistent with his neurocognitive baseline.

**TABLE 1:** The patient's ImPact testing scores of and the corresponding percentile rankings [1].

ImPact testing	Score	Percentile
Memory composite (verbal)	97	90th
Memory composite (visual)	92	92nd
Visual motor speed composite	43	84th
Reaction time composite	0.63	48th

## 4 Outcome and Follow Up

At that point, his only remaining symptoms were the reported constant bright flashes in his vision. MRI brain was ordered given the persistent visual symptoms and was negative for injury. Ophthalmology had evaluated and diagnosed him with visual snow syndrome. At this time his headaches had improved and he was back to pre‐injury baseline apart from the ongoing visual symptoms.

## 5 Discussion

**FIGURE 1:**
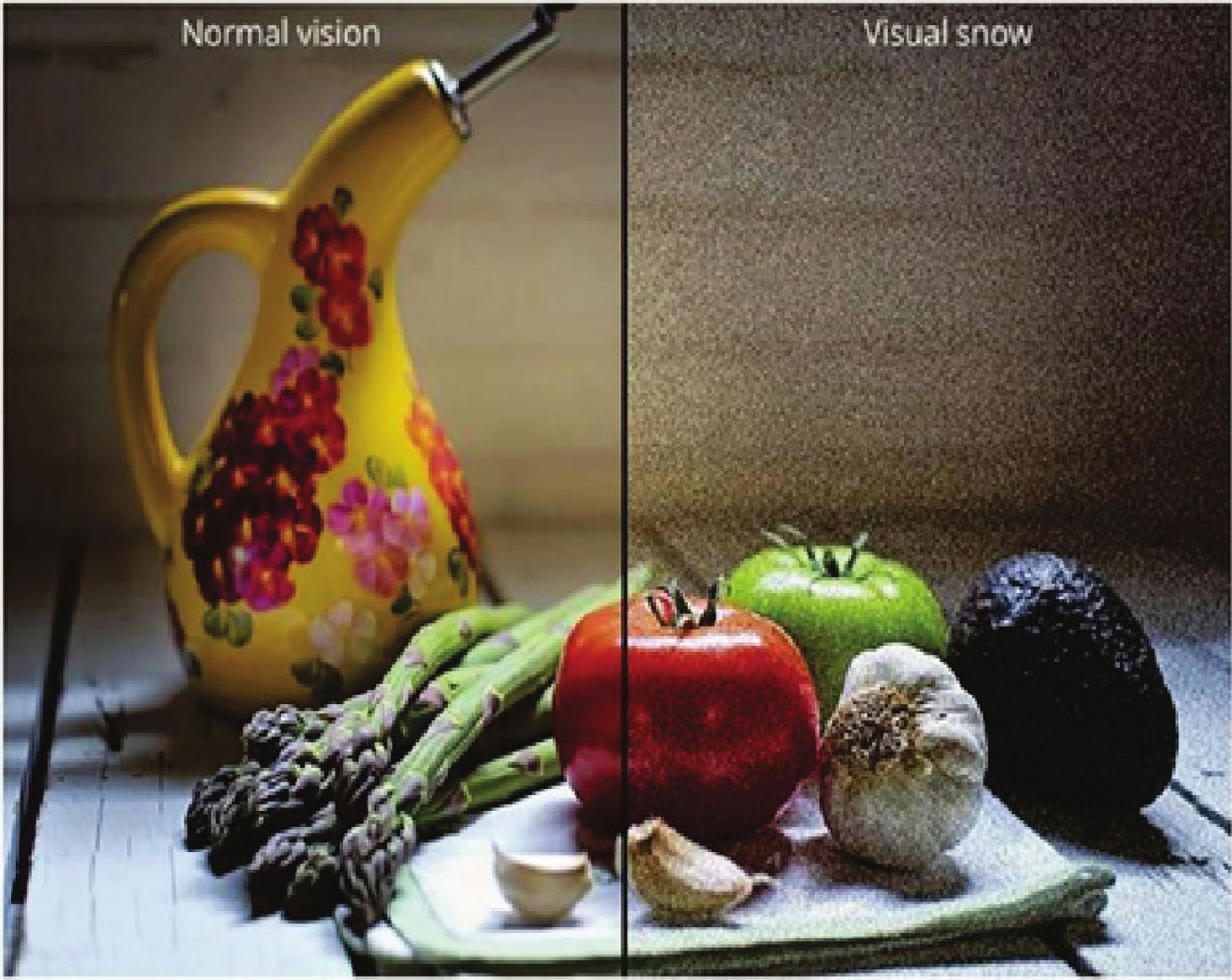
Visual representation of visual snow compared to normal vision [7].

Visual snow syndrome (VSS) is a visual disorder characterized by persistent flickering dots in all fields of vision resembling television static [2, 3, 4, 5], see Figure 1. Some patients also describe colored, transparent, or flashing dots in their fields of vision [2]. These dots have been described as black, brown, white, or translucent and last at least 3 months [2, 3, 4]. Patients also must report at least two of the following symptoms: photophobia, painful sensation to light, palinopsia, a visual after image, photopsia, flashes of light, and nyctalopia, impaired night vision [2, 3, 4, 6].

The incidence of VSS is not precisely known [2, 3]. VSS can affect adults and children and there have been a few studies suggesting a slightly higher prevalence among women [2, 3]. While the prevalence of migraines with visual aura in those with VSS is higher than the general population [3], VSS is a separate diagnosis with overlapping physiology [2, 3, 6]. There are case reports and literature describing VSS in patients who have sustained mTBIs [5] though none from a rehabilitation perspective. Increasing knowledge of VSS in the rehabilitation field is vital to form thorough differential diagnoses for vision changes following mTBI and provide recommendations for appropriate multidisciplinary workup and management of VSS. Patients have reported onset of symptoms after severe migraine attacks, use of certain medications such as levetiracetam, trauma, or infection [3, 6].

Research into pathophysiology has been more prevalent in the last 5 years and while the exact physiology of VSS is not yet known, there are several proposed mechanisms [3]. One proposed mechanism is that VSS is a cortical phenomenon [3]. Visual disorders due to localized deficits or hyperfunction in the V1/V2 areas of the occipital cortex can present with hallucinations similar to visual snow, and a lower threshold for occipital cortex excitability was seen in VSS patients compared to controls [3]. Recent studies have shown VSS patients have subtle, widespread anatomical differences with an increase in gray matter volume in the primary and secondary visual cortexes and V5 visual motion area, suggesting a more generalized biological basis for VSS rather than isolated occipital cortex involvement [3]. The thalamus could also contribute to VSS [3]. The lateral geniculate nucleus (LGN) receives visual sensory information from the retina, and the thalamic nuclei interpret this information and then present it to the cerebral cortex via the thalamocortical radiations [3]. Increased localized activity in the LGN could be responsible for VSS symptoms, and increased diffusion on MRI has been reported in thalamic radiations of VSS patients compared to controls [3].

VSS appears to be a nonprogressive condition though it can fluctuate in the severity of presentation [3]. VSS can be debilitating and lead to chronic disability for patients [3, 4] and thus, an honest discussion of the current research and limited treatment options should be had with patients [2]. Patient‐led support groups can be helpful [3]. In cases where VSS was brought on by an inciting event, such as a concussion, management of the underlying event may cause improvement of the VSS symptoms [3].

Proposed treatment options for VSS are as varied as the proposed pathophysiologic mechanisms. Specialized tinted glasses to reduce the perceived intensity of the visual snow, typically in the blue‐yellow color spectrum, have been helpful in subsets of patients, particularly those suffering from migraine, as it can reduce the cortical hyperactivity [3, 5]. Saccadic therapy can be used to address the perceived intensity of the palinopsia in order to recalibrate the system and allow for improved saccadic suppression [3, 5]. There have been conflicting reports on the efficacy of medications for treatment of VSS including benzodiazepines, lamotrigine, topiramate, and acetazolamide, with the highest success rate being lamotrigine helping in 8 out of 36 cases [2, 3].

## 6 Conclusion

Visual Snow Syndrome (VSS) can occur after a variety of triggers including mild traumatic brain injury (mTBI) and is very disruptive to patients' lives. Photopsia, bright flashes in the patient's field of vision, is a possible presentation of VSS though less common than the typically described “television static” vision changes. There is a lack of literature regarding the frequency of patients that present with photopsia as a primary symptom of VSS. It is vital for providers who evaluate patients who have sustained mTBIs to have knowledge of VSS to form complete differential diagnoses for vision changes following mTBI as well as knowledge of available treatment options to provide appropriate counseling and care.

## Author Contributions

**Katherine Hogan:** validation, visualization, supervision, resources, writing – review and editing. **Nathan Evanson:** validation, supervision, resources, writing – original draft, writing – review and editing. **Sarah Matthews:** conceptualization, methodology, writing – original draft, writing – review and editing, data curation, resources, visualization, project administration. **Brad Kurowski:** conceptualization, methodology, data curation, validation, supervision, writing – original draft, writing – review and editing, resources.

## Funding

The authors have nothing to report.

## Consent

Written informed consent was obtained from the patient for the publication of this case report.

## Conflicts of Interest

Sarah Matthews has nothing to disclose. Nathan Evanson's program of research has received financial support through research grants from the Department of Defense CMDRP Vision Research Program. He is on the editorial board of Archives of Physical Medicine and Rehabilitation. Katherine Hudson's program of research has received financial support through the American Academy of Optometry. Brad Kurowski's program of research has received financial support through competitively funded research grants from the National Institutes of Health and the Centers for Disease Control. All grant funding goes to the institution. He is on the board of governors for the American Congress of Rehabilitation Medicine in a voluntary (unpaid) capacity. He has received honorary recognition for editorial work.

## Data Availability

Data sharing not applicable to this article as no datasets were generated or analysed during the current study.
